# Effect of Succinylation on Oxidation–Aggregation of Low-Density Lipoprotein and Formation of Off-Flavors in Heated Egg Yolks

**DOI:** 10.3390/foods13213489

**Published:** 2024-10-30

**Authors:** Liuyang Ren, Cheng Sun, Ying Lv, Lokesh Kumar

**Affiliations:** 1 Beijing Laboratory for Food Quality and Safety, Key Laboratory of Agricultural Product Detection and Control for Spoilage Organisms and Pesticides, Food Science and Engineering College, Beijing University of Agriculture, Beijing 102206, China; 2 Department of Wine, Food and Molecular Biosciences, Lincoln University, Lincoln 7647, New Zealand

**Keywords:** egg yolk, heat treatment, succinylation, off-flavor, lipoprotein oxidation

## Abstract

This study examined the effect of succinylation on protein oxidation–aggregation and the formation of off-flavors in heated egg yolks (EYs). The sensory evaluation, content of volatile compounds, stability of low-density lipoprotein (LDL) particles, and oxidation of lipid and protein at six levels of succinylated EY (0.25%, 0.5%, 1%, 2%, 5%, and 10%, *w*/*w*) were determined. The results showed that the succinylated thermal EY’s concentration of volatiles and off-flavors was reduced. Oil exudation and lipid and protein oxidation decreased with the improved succinylation degree. Succinylation also reduced the LDL particle size and changed the secondary structure (decreased the β-sheets and increased the α-helices) of protein in LDL particles. Meanwhile, succinylation could effectively change the thermal oxidation–aggregation of LDL protein by introducing succinyl groups with negative charges, thus increasing the stability of LDL particles in succinylated EY during heating. These results further revealed the relationship between the oxidation–aggregation of LDL and the formation of off-flavors in heated EY. These results also help improve the flavor quality of heat-treated EY and expands the application scope of egg products.

## 1. Introduction

Rich in protein, lecithin, minerals, and vitamins, egg yolk (EY) plays a vital role in neurotransmission, the development of the brain, and the integrity of the bones [1]. Meanwhile, because of its superior heat coagulation and emulsification capabilities, it is used in various foods (creams, cakes, omelets, and confectionery) [2]. Thermal treatments, such as pasteurization and spray drying, are frequently employed to process egg products. However, heat treatment leads to an unpleasant off-flavor of EY (a fishy, marine, or shellfish smell), thus affecting egg products’ sensory qualities [3,4].

Yolk is a complex matrix containing 16% proteins, 27% lipids, and other components [1]. Low-density lipoprotein (LDL) particles (ranging in size from 17 nm to 60 nm) are spherical nanoparticles with a lipid core surrounded by a monofilm of phospholipids and proteins. The lipid core is composed of triglycerides and cholesterol esters [5,6]. About 90% of yolk lipids, which include 26% phospholipids and 74% neutral lipids, and 50% of yolk proteins are made up of these particles [7]. During the processing and storage, unsaturated fatty acids are exposed to light, heat, or air, which causes quick and widespread oxidation [8], producing hydroperoxides and, eventually, off-flavor chemicals [9,10]. For heated egg yolk, the key off-flavor components have been identified as 1-octen-3-ol, 2-pentylfuran, hexanal, (*E*)-2-octenal, and (*E, E*)-2,4-decadienal, which are oxidative products of nonfatty acids [4]. Moreover, natural antioxidants have been reported to inhibit the formation of unacceptable flavors resulting from the degradation of lipids and proteins in egg yolk [11]. Furthermore, it has been reported that the instability of LDL particles leads to the leakage of lipids and, thus, the formation of off-flavors in thermal egg yolk [4,12]. However, the methods of maintaining the stability of thermal LDL particles are unclear.

On the other hand, proteins are crucial for imparting O/W emulsion stability and are widely used in the food industry [13]. Whey proteins are commonly used as emulsifiers in food systems to stabilize oil droplets. When subjected to heat treatment above 70 °C, these proteins unfold and aggregate, contributing to the coalescence and flocculation of emulsion droplets [14]. Protein oxidation can lead to protein cross-linking and amino acid side chain modification or fragmentation [15], resulting in a decrease in the interfacial and emulsifying properties of proteins. After excessive oxidation, the rice bran protein forms aggregates, the structure of interfacial adsorption protein changes, the protein flexibility weakens, and the protein emulsion stability is, thus, reduced [16,17]. Nonetheless, the inhibition of black bean protein oxidation facilitates the improvement in the thermal stability of protein emulsion [18]. In a manner similar to oil-in-water (O/W) emulsions, oxidative aggregation and changes in the secondary structure of proteins in LDL particles—characterized by an increase in β-sheets and a decrease in α-helices—results in a reduction in the particles’ thermal stability in egg yolk [12].

Succinylation is a common chemical modification which occurs through the interaction of succinyl groups with amino, hydroxyl, or thiol groups that significantly alters the charged state of proteins [19]. Succinylation enhances the solubility and decreases the particle size of milk proteins by preventing the formation of protein aggregates [20]. The introduction of succinyl groups enhances protein electronegativity, thereby increasing the molecular weight and decreasing the surface hydrophobicity of oat proteins [21]. Succinylation improves the stability of soy proteins after heating by suppressing their thermal aggregation [19] and also increases electrostatic repulsion and decreases protein cross-linking, thus improving the thermal stability of egg white protein [22]. These studies demonstrate that succinylation modification affects protein characteristics such as the net negative charge, molecular structure, and surface hydrophobicity, all of which have an influence on the thermal aggregation of proteins. 

Nevertheless, the behavior of succinylated EY proteins during thermal aggregation has not been extensively studied. Moreover, it is still unknown how succinylation affects the stability of LDL particles and the development of off-flavors in thermally treated EY. In this study, thermal protein aggregation was first analyzed using gel electrophoresis, surface hydrophobicity, and Fourier-transform infrared spectroscopy (FTIR). Next, the confocal laser scanning microscopy (CLSM), particle size, and zeta potential were used to evaluate the stability of LDL particles. Moreover, the effect of succinylation on the off-flavor compounds of heated EY was investigated by gas chromatography–mass spectrometry (GC–MS) and sensory evaluation. As a result, our work offers a foundation for a deeper comprehension of the connection between LDL protein oxidation–aggregation and the formation of off-flavor compounds in thermally treated egg yolk. It could provide guidelines for proposing appropriate methods to inhibit the unpleasant flavor of egg products.

## 2. Materials and Methods

### 2.1. Materials

Duck eggs, 5–10 days post-laying, were purchased from the Beijing Changping Huilongguan market.

### 2.2. Preparation of Samples with Different Succinylation Degrees (SDs)

Preparation of succinylated EY: Eggs were broken, and egg whites were separated to isolate the yolks. Egg yolks were then combined with water at a 1:1 (*w*/*w*) ratio and stirred to obtain a uniform dispersion. Succinylated EY samples were prepared following the method outlined by Ren et al. [23]. Succinic anhydride (SA) was added to the EY dispersion at concentrations of 0.25%, 0.5%, 1%, 2%, 5%, and 10% (*w*/*w*) to yield succinylated EY with varying degrees of succinylation. A control sample was similarly prepared, omitting SA. All samples were stored at 4 °C after the succinylation. 

Preparation of succinylated EY at varying heating temperatures: Succinylated EY samples, with SA concentrations of 0, 0.5%, and 5% (*w*/*w),* were prepared in plastic syringes following the method of Ren et al. [23]. The samples were heated at 65 °C, 70 °C, and 90 °C for 35 min, then cooled to 25 °C and stored at 4 °C for 12 h. Succinylated EY samples heated at 65 °C and 70 °C were designed for analyses of oil exudation, microstructure, and lipid or protein oxidation, while those heated at 90 °C were used to evaluate volatile compounds and conduct sensory assessments.

Preparation of succinylated LDL particles with different heating temperatures: Succinylated EY samples, with SA concentrations of 0, 0.25%, 0.5%, 1%, 2%, 5%, and 10% (*w*/*w*), were heated at 60 °C for 35 min. This succinylated EY was used to prepare LDL particles according to Ren et al. [12]. EY and 0.17 M NaCl solution were mixed at a ratio of 1:1 (*v*/*v*) and stirred for 1 h at 4 °C. Subsequently, these EY samples were centrifuged at 15,000× *g* for 15 min at the same temperature. The supernatant (plasma) was separated from the precipitate (particles). The plasma, with a pH level of 6.0 ± 0.1, was then combined with 5% PEG, which had a polymerization degree of 4,000, and 0.02% Vc to prevent the oxidation of LDL. This mixture was stirred for 1 h at 4 °C and then centrifuged at 22,000× *g* for 15 min at 4 °C. The floating material at the top, which consisted of purified LDL particles, was collected and prepared for further analysis. Thermal LDL particles with succinylation modification were used for electrophoresis, secondary structure, and physicochemical properties analysis.

### 2.3. Determination of Modification Degree

The amount of modified amino group (degree of N-succinylation) was calculated by measuring the free amino group content with the o-phthaldialdehyde method [24]. The degree of N-succinylation (%) was expressed by the percent decrease in the content of free amino groups in succinylated samples compared with the native sample. The degree of S-succinylation for sulfhydryl groups was determined using 5,5-dithiobis-2-nitrobenzoic acid [25]. The degree of S-succinylation (%) was determined by measuring the percent reduction in sulfhydryl groups content in the succinylated samples compared to the native sample.

### 2.4. Sensory Evaluation

The succinylated EY was poured into 20 mL of plastic syringes (2 cm in diameter) with the needle part removed. The lower and upper ends were sealed with rubber pistons and plastic wrap, respectively. The plastic syringes were heated at 90 °C for 35 min to obtain EY gels. The gels were cooled at room temperature and kept at 4 °C for 12 h. The sensory properties of samples were evaluated by nine trained panelists (including five female and four male postgraduate students aged 22–26) recruited from Beijing University of Agriculture. All panelists were trained using various flavor standards to ensure that they could accurately recognize the off-flavor intensity of the egg samples. The panelists were subjected to a ranking test with a series of five aqueous solutions of hexanal, (E)-2-octenal, (E, E)-2,4-decadienal, 1-octen-3-ol, and 2-pentylfuran. Nine panelists were selected, and five 2 h training sessions were held in the sensory room at 23 ± 2 °C. During the focused training, panelists evaluated and discussed an array of heated EY gels. These samples included gels prepared by mixing egg yolk liquid and water in different proportions and heating them. Additionally, the panelists were also exposed to a series of tests in which five key off-flavor substances were added in varying concentrations to odorless EY matrices. The artificial odorless matrix was prepared according to the method of Ren et al. [4].

Samples were removed from refrigeration 1 h prior to evaluation and were well-crushed. A 10 g portion of the sample was dispensed into cups labeled with a three-digit random code and covered with a lid. Panelists independently assessed the off-flavor intensity of the seven samples in a randomly balanced order. Panelists took a 2 min break between each sample and then a 10 min break between each replicate. Samples were evaluated in triplicate. Assessors used a five-point scale (0 (none); 1 (very weak); 2 (weak); 3 (medium); 4 (strong); and 5 (very strong)) to evaluate the off-flavor intensity of heated EY with different SDs.

### 2.5. Analysis of Volatile Compounds

Volatile compounds of succinylated EY were extracted and measured using the headspace solid-phase microextraction–GC–MS method as described by Ren et al. [4]. Among these volatiles, the key off-flavor components (hexanal, (E)-2-octenal, (E, E)-2,4-decadienal, 1-octen-3-ol, and 2-pentylfuran) were quantified by the GC–selected ion monitoring method.

### 2.6. Confocal Laser Scanning Microscopy (CLSM)

The structure and morphology of succinylated EY samples at different heating temperatures (65 °C and 70 °C) were detected using CLSM (ZEISS LSM-980, Carl Zeiss AG, Jena, Germany). Staining involved mixing 200 μL of the sample with 20 μL of Nile Red (0.1%, *w*/*v*) to observe lipid droplets and with 10 μL of Fast Green solutions (1.0%, *w*/*v*) to observe proteins. The excitation wavelengths of Nile Red and Fast Green were 488 nm and 633 nm, respectively. Samples were observed under the 63× oil immersion objective lens.

### 2.7. Oil Exudation from Thermally Succinylated EY

The oil exudation (%) was determined based on the method of Li et al. [26] with some modifications. A 35 mL portion of n-hexane/2-propanol (3:2, *v*/*v*) was added to 3 g of yolk samples and then homogenized at 5000 rpm for 30 s (T18BS25, IKA, Wertheim, Germany). After filtration, the filtrate was evaporated in a water bath (55 °C) and then dried at 105 °C to a constant weight, and the residue was weighed as total lipid content. The yolk (5 g) was incorporated into deionized water (25 mL) and homogenized (5000 rpm, 30 s). After centrifugation (11,200× *g*, 30 min), the free fat was distributed in the supernatant. A 25 mL portion of n-hexane/2-propanol (3:2, *v*/*v*) was added to the supernatant. The mixture was homogenized under the same conditions and then filtered. The filtrate was evaporated in a water bath (55 °C) and then dried at 105 °C to a constant weight, and the residue was weighed as free lipid content. The oil exudation (%) was calculated by the ratio between the free lipid content and the total lipid content.

### 2.8. Lipid and Protein Oxidation

Lipid oxidation was investigated by determining thiobarbituric acid reactive substances (TBARS). The samples were succinylated EY (with the addition of SA at 0, 0.5%, and 5%, *w*/*w*) heated at 65 °C and 70 °C. A 10 g portion of the sample was homogenized with 20 mL of trichloroacetic acid (TCA, 15%, *w*/*v*)–TBA (0.375%, *w*/*v*)–HCl (0.25 mol/L) solution at 3500 rpm for 2 min. The homogenate was heated in a water bath (100 °C, 15 min) and cooled to 25 °C, and then centrifuged at 3000× *g* for 10 min. The absorbance of the supernatant was measured at 532 nm by UV–Vis spectrophotometer (T6 New Century, Beijing General Instrument Co., Ltd., Beijing, China). TBARS was calculated from the standard curve of malonaldehyde (MDA), the decomposition product of tetraethoxypropane, and the result was expressed as mmol MDA/mg homogenate. Protein oxidation was investigated by determining total carbonyl content. The 10 g portion of the sample was incorporated into 20 mL of 1-butanol/di-isopropyl ether (2:3, *v*/*v*) to remove lipids that interfered with carbonyl content determination. The measure method of carbonyl content was performed according to Ren et al. [12].

### 2.9. Sodium Dodecyl Sulphate–Polyacrylamide Gel Electrophoresis (SDS–PAGE)

The molecular weight of protein subunits in succinylated samples was determined using SDS–PAGE as reported by Tao et al. [27] with modifications. Acrylamide stacking and separating gels were 5% and 12%, respectively. The samples were diluted in dissociation buffer (1:5, *v*/*v*), containing 0.25 M Tris-HCl (pH 6.8), 0.5% (*w*/*v*) bromophenol blue, 50% glycerol, 10% SDS solution, and 5% β-mercaptoethanol to obtain reduced SDS–PAGE patterns. Before electrophoresis, the mixtures were heated at 100 °C for 3 min and then centrifuged at 10,000 *g* for 5 min. The electrophoresis was performed at 20 mA in the stacking gel and 40 mA in the separating gel until the tracking dye reached the bottom of the gel. The gels were scanned using a gel imaging analysis system (BG-gdsAUTO520, Baygene Biotech Co., Ltd., Beijing, China).

### 2.10. FTIR

The prepared LDL particles were frozen with liquid nitrogen (99.99%) and then dried at −60 °C for 24 h with an ALPHA 1–4 LSC freeze-dryer (Martin Christ Gefriertrocknungsanlagen GmbH, Ltd., Osterode, Germany). The FTIR spectra of proteins in native and succinylated LDL particles were analyzed using a Thermo Scientific Nicolet iS5 FTIR spectrometer (Thermo Fisher Scientific, Waltham, MA, USA). The samples were scanned in the range of 4000–400 cm^−1^ with 32 scans. The region of 1700–1600 cm^−1^ was analyzed with the PeakFit software (version 4.12, Sea-Solve Software Inc., Framingham, MA, USA). 

### 2.11. Determination of Physicochemical Properties

Intrinsic fluorescence emission spectra of the EY were investigated according to Wan et al. [19]. Surface hydrophobicity was measured with 1-anilino-8-naphthalenesulfonate (ANS) [28]. A Zetasizer Nano ZS 90 (Malvern Instruments, Southborough, UK) was used to determine the LDL particle size and zeta potential of all succinylated EY samples. 

### 2.12. Statistical Analysis

The data were analyzed and processed using SPSS 25.0 (IBM Inc., Armonk, NY, USA). The FTIR spectra of proteins in succinylated EY were evaluated in duplicate, and all other experiments were conducted in triplicate. Mean values were compared using one-way analysis of variance (ANOVA), and the Duncan multiple range test was used to determine the significant differences between groups (*p* < 0.05).

## 3. Results and Discussion

### 3.1. Succinylation of EY

**Figure 1:**
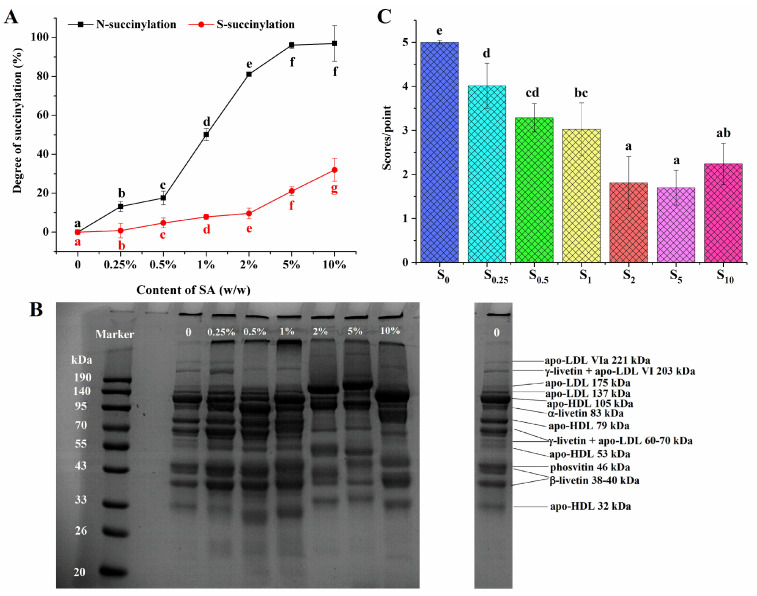
N-succinylation (in black) and S-succinylation (in red) degrees of modified EY with different succinic anhydride (SA) additions (**A**); SDS–PAGE profiles of native and succinylated EY protein (**B**). Proteins were identified based on SDS–PAGE profiles by Guilminean et al. [30]; sensory evaluation scores of off-flavor in thermally succinylated EY (**C**). S_0_, S_0.25_, S_0.5_, S_1_, S_2_, S_5_, and S_10_ represent succinylated EY obtained at the anhydride addition of 0, 0.25%, 0.5%, 1%, 2%, 5%, and 10% (*w*/*w*), respectively. Bars with different letters mean significant difference (*p* < 0.05).

The succinylation reaction mainly occurs at the ε-amino group of lysine and partially at the sulfhydryl groups of proteins in egg yolk [23]. Figure 1A showed the effect of the SA content on the N-succinylation and S-succinylation degrees of EY. The degree of N-succinylation rapidly increased to 96.14 ± 1.53% when the addition of SA went from 0 to 5% (*w*/*w*). After that, the N-succinylation degree remained unchanged. This phenomenon was consistent with the findings of Wang et al. [29] that the N-succinylation degree of Antarctic krill proteins increased linearly as the ratio of the anhydride–amino group content increased, while the degree of succinylation was not further increased at the ratio of 0.5 g/g. When most of the amino groups were succinylated, the sulfhydryl groups reacted with SA subsequently. At anhydride ratios of less than 2% (*w*/*w*), the degree of S-succinylation increased slowly (from 0.79 ± 3.73% to 9.60 ± 2.76%), whereas, at high levels of anhydride content (above 2%, *w*/*w*), the degree of S-succinylation improved remarkably (from 9.60 ± 2.76% to 31.97 ± 5.77%, *p* < 0.05). Similar to the results observed with succinylated kidney bean protein, the degree of S-succinylation persistently increased with the enhancement of the anhydride ratio [25]. 

The SDS–PAGE patterns of native and succinylated EY were given in Figure 1B. The identification of all major LDL proteins and livetins was consistent with the report of Guilminean et al. [30]. As shown in Figure 1B, compared with the native succinylated EY, the protein electrophoresis depicted almost no obvious difference when the addition amount of SA was 0.25% (*w*/*w*). When the anhydride content rose to 0.5% and 1% (*w*/*w*), the intensity of γ-livetin and the apo-LDL VI 203 kDa band became weaker, and the 83 kDa (α-livetin) band turned darker; the apo-LDL VIa 221 kDa band almost disappeared. Moreover, the bands at 33–190 kDa also changed for samples with SA additions of 2–10% (*w*/*w*) in comparison with the unsuccinylated EY. The molecular weight of oat and mung bean proteins had been reported to undergo significant changes following succinylation modification [21,31]. The SDS-PAGE results indicated that succinylation causes significant structural changes in EY proteins, especially at higher SA concentrations. The disappearance and intensity changes of specific protein bands suggested protein modifications.

### 3.2. Off-Flavors of Heated EY with Succinylation Modification

Figure 1C indicated that the off-flavor score of heated EY decreased as succinylation increased. When the addition of anhydride was 2% (*w*/*w*), the off-flavor score declined significantly compared to samples with SA additions of 0, 0.25%, 0.5%, and 1% (*w*/*w)*. However, there was no significant difference for succinylated thermally treated EY (S_2_, S_5_, and S_10_) (*p* > 0.05). It seemed that the concentration of SA had an influence on the sensory properties of heat-treated yolk. 

In heated EY, hexanal, (E)-2-octenal, (E, E)-2,4-decadienal, 1-octen-3-ol, and 2-pentylfuran were the key off-flavor compounds [4]. In Table 1, the concentration of (E, E)-2,4-decadienal and 2-pentylfuran decreased for the heat-treated EY of S_0.5_, whereas that of hexanal, (E)-2-octenal, and 1-octen-3-ol declined for the S_1_ samples. Additionally, the content of hexanal, (E)-2-octenal, (E, E)-2,4-decadienal, and 2-pentylfuran in the S_10_ EY sample increased with an excessive anhydride addition, showing no significant difference compared with that of S_5_ (*p* > 0.05). Meanwhile, the concentration of 1-octen-3-ol in S_10_ was significantly higher than that in S_5_ (30.91 > 15.87, *p* < 0.05). It seems that, when the addition of succinic anhydride exceeds 5%, it may have a minimal impact on reducing the off-flavor. This suggested that increasing the concentration beyond this threshold did not significantly influence the flavor profile, indicating a possible saturation point in the effectiveness of succinic anhydride in reducing undesirable sensory qualities. However, all succinylated samples showed reduced concentrations of key off-flavor substances compared to the control. The data were consistent with Figure 1C, which presented that succinylation treatment could inhibit the production of off-flavors in heated EY. 

### 3.3. Oil Exudation in Thermally Succinylated EY

It was reported that the concentration of off-flavors and volatile compounds increased significantly when the EY was heated at 60 °C–65 °C [12]. Additionally, the structure of LDL particles was partly destroyed, and lipid leakage was observed at this temperature [12]. The effect of succinylation on the lipid distribution and stability of LDL particles was investigated. The oil exudation of thermally the succinylated EY (S_0.5_ and S_5_) was then measured and shown in Table 2. The oil exudation of the EY heated at 65 °C was lower than that at 70 °C, regardless of the amount of anhydride addition. Moreover, oil exudation declined significantly as the SA concentration increased (*p* < 0.05). This indicated that succinylation significantly reduced the oil exudation of the thermal EY and improved the stability of LDL particles.

**Figure 2:**
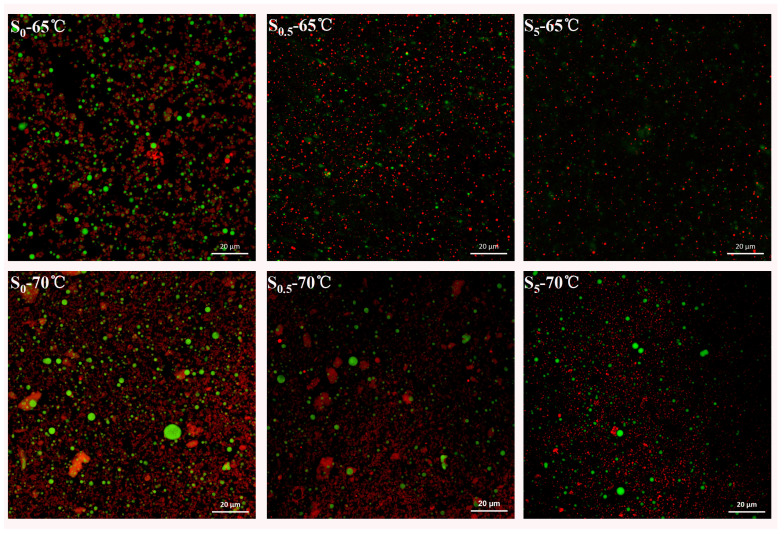
Confocal laser scanning microscopy (CLSM) of succinylated EY heated at 65 °C and 70 °C (lipids appear in red and proteins in green). S_0_, S_0.5_, and S_5_ represent succinylated EY obtained at the anhydride addition of 0, 0.5%, and 5% (*w*/*w*), respectively. The scale bar in all images is 20 µm.

As shown in Figure 2, large lipid droplets (indicated by red points) were clearly visible in the unsuccinylated samples (S_0_—65 °C and S_0_—70 °C), with the 70 °C-treated samples exhibiting a higher density of lipid distribution compared to those treated at 65 °C. This suggested that the higher temperature caused a more significant disruption to LDL particles, leading to increased lipid leakage. Conversely, as the concentration of SA increased (S_0.5_ and S_5_), the number of oil droplets decreased, aligning with the oil exudation results presented in Table 2. These findings indicated that succinylation effectively reduced the heat-induced lipid leakage in egg yolk by stabilizing the LDL particles and reducing oil release during thermal processing.

### 3.4. Lipid and Protein Oxidation in Thermally Succinylated EY

As shown in Table 2, the TBARS value decreased with the increasing addition of SA, regardless of whether it was heated at 65 °C or 70 °C. The unsaturated fatty acids in triacylglycerols decomposed during lipid oxidation to form small, volatile molecules that produced the off-flavors associated with oxidative rancidity [32]. The results in Figure 1C and Table 1 indicated that the key components of off-flavors for the succinylated EY decreased. This indicated that succinylation may have prevented the formation of off-flavors from lipid oxidation in heated egg yolk.

Table 2 also depicted that, with an increase in SA concentration, the carbonyl content of EY heated at 65 °C decreased significantly from 0.52 ± 0.05 nmol/mg protein to 0.31 ± 0.02 nmol/mg protein (*p* < 0.05). A similar trend was also found for the thermally treated egg yolk (70 °C) with succinylation treatment. The formation of carbonyl groups is considered one of the most obvious changes after protein oxidation [33]. From the data on carbonyl content, it was observed that succinylation could decrease the oxidation of proteins in heat-treated EY.

During the thermal process, egg yolk generates various oxidation products, which can form oxidized side-chain groups and potentially cause protein aggregation or degradation [34,35]. LDL particles contain a lipid core stabilized by a monolayer of phospholipids and proteins. As a major component of O/W emulsions in EY, it was reported that the structural change of LDL protein, resulting from thermal oxidation, could reduce the stability of LDL particles [12]. This phenomenon was also shown in Figure 2. Furthermore, the effect of succinylation modification on the protein structure and stability of LDL particles was discussed.

### 3.5. Zeta Potential and Particle Size of Heated Succinylated LDL Particles

**Figure 3:**
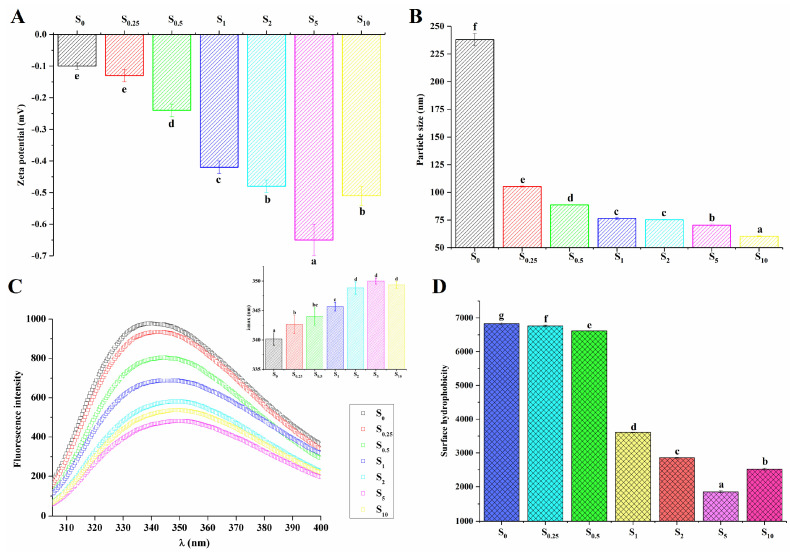
The zeta potential (**A**) and particle sizes (**B**) of LDL particles in heated EY modified by succinylation; and intrinsic fluorescence emission spectra, λ_max_ (**C**) and surface hydrophobicity (**D**) of protein from LDL particles in heated EY with succinylation treatment. S_0_, S_0.25_, S_0.5_, S_1_, S_2_, S_5_, and S_10_ represent succinylated EY obtained at the anhydride addition of 0, 0.25%, 0.5%, 1%, 2%, 5%, and 10% (*w*/*w*), respectively. Bars with different letters mean significant difference (*p* < 0.05).

The zeta potential was used to evaluate the colloidal stability of proteins. In Figure 3A, compared with the unsuccinylated sample (S_0_), the absolute value of the zeta potential for all LDL particles with the succinylation modification improved (*p* < 0.05), thus confirming that the negative charges were introduced to the protein by succinyl groups. Significant differences also existed among the succinylated LDL particles (S_0.25_, S_0.5_, S_1_, S_2_, and S_5_, *p* < 0.05). The increase in the zeta potential value with an increasing modification degree was also found in succinylated kidney protein isolate and soy protein isolate [19,25]. As shown in Figure 3A, negative charges were introduced onto the protein surface by succinylation. It was reported the negative charges produced an electrostatic repulsion force, thereby preventing the coalescence of droplets with one another [19]. Proteins that adsorb at the oil–water interface typically stabilize emulsions by forming electrically charged interfacial films [36]. These films prevent droplet flocculation by promoting electrostatic repulsion, especially when the system has a higher surface charge [36], as is the case with succinylation. Hence, as illustrated in Figure 3B, the size of LDL particles decreased with the increase in succinylation degree, which was consistent with the results obtained by CLSM (Figure 2). Particles with a small size and high zeta potential conferred stability, which could make them resistant to aggregation [11]. Thus, succinylation was beneficial in maintaining the thermal stability of LDL particles. 

### 3.6. FTIR of Proteins in Heated Succinylated LDL Particles

The amide I region with wavelengths between 1700 and 1600 cm^−1^ is the most informative part of the FTIR spectrum for the protein secondary structure [37]. The secondary structure was used to evaluate the effect of succinylation on the conformational changes in protein within LDL particles. According to the relevant research, the absorption band of β-sheets, α-helices, β-turns, and random coils was assigned to 1600–1639 cm^−1^, 1651–1660 cm^−1^, 1661–1700 cm^−1^, and 1640–1650 cm^−1^, respectively [38]. In Table 3, the content of β-sheets and α-helices in S_0_ was 22.60 ± 1.01% and 30.92 ± 0.58%, respectively. With the increasing addition of SA (0.25–10%, *w*/*w*), the β-sheet content reduced from 21.91 ± 3.08% to 13.77 ± 0.89%, whereas the α-helix content rose from 29.54 ± 1.23% to 42.64 ± 0.05%. The ratio of the β-turn and random coil did not present any regular change as the SA content increased. It was reported that amphipathic α-helixes with hydrophilic and hydrophobic regions helped to preserve the stability of apoprotein structures [5]. The evident improvement of the α-helix for the succinylated samples explained the data in Figure 2 and Figure 3B, indicating that succinylation could prevent oil droplets from coagulating and enhance the stability of LDL particles. 

### 3.7. Intrinsic Fluorescence Emission Spectra and Surface Hydrophobicity

As shown in Figure 3C, the drastic reduction in the fluorescence intensity of protein in LDL particles was related to the increasing succinylation degree (S_0_, S_0.25_, S_0.5_, S_1_, S_2_, and S_5_), while the intrinsic fluorescence intensity of S_10_ increased in comparison to S_5_. It was reported that the changes in intrinsic fluorescence intensity were caused by the alteration of the spatial conformation around the Trp residues [39]. It seemed that succinylation changed the tertiary or quaternary structure of proteins in LDL particles. Furthermore, the maximum fluorescence emission wavelength (λ_max_) moved to a larger value (red shift) as the succinylation degree improved (S_0_, S_0.25_, S_0.5_, S_1_, and S_2_) and subsequently remained steady for S_2_, S_5_, and S_10_ (Figure 3C inside). When proteins underwent conformational changes, the exposure of Trp increased, which led to λ_max_ shifting towards a higher wavelength [21]. The red shift in λ_max_ indicates a greater exposure of hydrophobic amino acid to the polar solvent environment. The result suggested that the hydrophobic interactions within protein molecules were altered due to succinylation. 

The surface hydrophobicity of protein was evaluated using the enhanced fluorescence induced by the ANS binding to the hydrophobic region of the protein surface. As seen in Figure 3D, the surface hydrophobicity of the modified samples reduced significantly with the increasing modification degree (*p* < 0.05). On one hand, the stronger repulsive forces generated by the increased density of negative charges (introduced by succinic anhydride) prevented ANS from accessing the hydrophobic clusters of modified protein [19,23]. Additionally, the intrinsic fluorescence emission results revealed structural transformations in the protein following modification (Figure 3C). The modification of the protein structure resulted in the enhanced burial of nonpolar amino acids within the hydrophobic core, while polar amino acids were redistributed on the molecular surface. This conformational shift reduced the accessibility of ANS to the hydrophobic regions of the protein, leading to a significant decrease in the surface hydrophobicity of the modified egg yolk. The observed reduction in surface hydrophobicity is indicative of the structural alterations in the protein, which are likely responsible for the reduced binding affinity of hydrophobic probes, as described by Shilpashree et al. [20]. 

### 3.8. Changes of Protein Subunits in Succinylated LDL Particles

**Figure 4:**
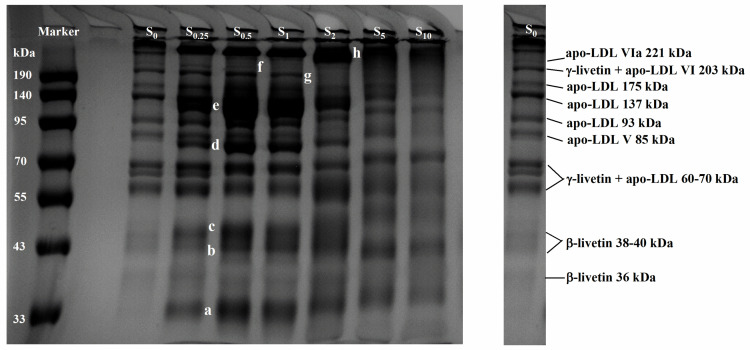
SDS–PAGE profiles of protein from LDL particles in heated EY with succinylation treatment. S_0_, S_0.25_, S_0.5_, S_1_, S_2_, S_5_, and S_10_ represent succinylated EY obtained at the anhydride addition of 0, 0.25%, 0.5%, 1%, 2%, 5%, and 10% (*w*/*w*), respectively. Bands a–h (a, b, c, d, e, f, g, and h) represent a variety of proteins with different molecular weights.

The effect of the SA addition (0–10%, *w*/*w*) on the molecular weight distribution of protein in LDL particles was shown in Figure 4. Compared to the unsuccinylated sample (S_0_), new protein bands emerged, including a band around 34 kDa (marked as a), while additional bands at 80 kDa and 110 kDa (marked as d and e) were observed in samples S_0.25_, S_0.5_, S_1_, and S_2_. Moreover, the intensity of bands at the position of b–c (approximately 44–47 kDa) increased with the rising SA addition (S_0.25_–S_1_), which were identified as β-livetin 38–40 kDa. A single, high-molecular-weight band at the top of the gel (h) also increased in intensity, indicating the accumulation or aggregation of heavier protein complexes. Additionally, the prominent proteins in egg yolk, specifically those with molecular masses of 205 kDa (γ-livetin + apo-LDL VI, 203 kDa) and 217 kDa (apo-LDL VIa, 221 kDa), became nearly undetectable when SA was added at concentrations of 2%, 5%, and 10%. This suggested that the higher levels of succinylation significantly disrupted the integrity of these high-molecular-weight proteins, leading to their degradation or aggregation during the modification process. Thus, succinylation appeared to significantly alter the aggregation behavior of proteins in LDL particles during heat treatment. It seemed that the introduction of succinyl groups altered the charge distribution and surface hydrophobicity of the proteins, which might have reduced or promoted the thermal aggregation of proteins within LDL particles. 

Oxidation reactions were reported to have an impact on the charges and protein tertiary structure, resulting in changes in protein properties such as solubility and emulsifying ability (due to aggregation or complex formation) [40,41]. The proteins in egg yolk undergo significant oxidative reactions during thermal processing [42]. In our previous studies, EY was thermally treated across a range of temperatures (25 °C to 90 °C); it was demonstrated that higher temperatures (55–65 °C) facilitated the oxidation of proteins and lipids, while simultaneously leading to the formation of high-molecular-weight proteins. This indicated that protein oxidative aggregation, specifically involving LDL proteins and livetin within LDL particles, occurred around 60–65 °C [12]. However, succinylation decreased the carbonyl content and TBARS concentration of the heated egg yolk (Table 2). Additionally, the SA addition reduced the oil extrusion for EY heated at 65 °C and 70 °C (Figure 2 and Table 2), thus improving the thermal stability of LDL particles. Wan et al. [19] found that the electrostatic repulsion of succinyl groups was responsible for the suppression of thermal aggregation in soy protein isolate. Heat treatment caused the exposure of the hydrophobic groups within proteins, and the hydrophobic interaction between these groups was a main driver of protein aggregation [19]. Thus, the decrease in surface hydrophobicity caused by succinylation (Figure 3D) may have been the reason for the inhibition of protein aggregation, as shown in the SDS–PAGE. Moreover, the observed increase in the intensity of the high-molecular-weight protein band with the added SA suggested the formation of larger protein aggregates (Figure 4: the high-molecular-weight band at the top of the gel (h), which was indicative of protein oxidation and aggregation processes). In the context of thermal processing, succinylation induces structural changes in proteins, leading to the exposure of reactive amino acids like cysteine or lysine, which, in turn, promote the formation of disulfide bonds and other covalent cross-links, contributing to aggregation. Studies showed that heat treatment combined with succinylation accelerated the denaturation of egg yolk proteins, facilitating protein–protein interactions that resulted in the formation of higher-molecular-weight aggregates.

As pointed out in previous research, proteins play an important role in protecting lipophilic compounds from oxidation and acting as a physical barrier by forming a thick interfacial layer, thus preventing them from degradation and the generation of large-size droplets [43]. In egg yolk, LDL protein (combined with phospholipid) forms an interfacial layer (the LDL particle membrane) to protect lipids from being oxidized. However, our previous results showed that, during heat treatment, the oxidation–aggregation of LDL protein contributed to the destruction of particles [12]. The oil was released from the LDL particles, and then lipid oxidative compounds were formed and identified as off-flavors in heated egg yolk [12]. In this study, succinylation increased the stability of LDL particles by changing the protein oxidation–aggregation behavior (Figure 4) during heat treatment. The oil leakage from LDL particles and lipid oxidation were effectively controlled. Thus, the number of volatile substances derived from the oxidation products of protein and lipid was greatly reduced. The off-flavors of thermally treated EY were then inhibited by succinylation.

## 4. Conclusions

This study demonstrated that succinylation effectively inhibited the formation of off-flavors in heat-treated egg yolk. Increasing the degree of succinylation significantly reduced both the sensory evaluation scores and the concentration of volatile compounds, indicating an improvement in flavor quality. Succinic anhydride was shown to prevent oil leakage from LDL particles and to suppress lipid and protein oxidation at temperatures of 65 °C and 70 °C. This modification enhanced the thermal stability of LDL particles by increasing the absolute value of the zeta potential and reducing the particle size. Additionally, succinylation altered the surface hydrophobicity and secondary structure of proteins in LDL particles and influenced their oxidation–aggregation behavior during heating. These findings highlighted the crucial role of succinylation in preserving the stability of LDL particles and the flavor quality in thermally processed egg yolk. However, since succinic anhydride is not edible, further research is required to identify alternative methods for reducing off-flavors by inhibiting LDL protein oxidation and aggregation, thereby enhancing the edible quality of egg products.

## Figures and Tables

**Table 1:** The concentrations of key off-flavor volatile compounds in succinylated egg yolk.

	Concentration (ng/g)				
	Hexanal	(E)-2-Octenal	(E, E)-2,4- Decadienal	1-Octen-3-ol	2-Pentylfuran
S_0_	120.86 ± 2.85 ^c^	23.89 ± 1.11 ^b^	2.43 ± 0.62 ^c^	71.03 ± 1.60 ^d^	0.80 ± 0.03 ^d^
S_0.25_	117.10 ± 6.36 ^c^	23.40 ± 2.48 ^b^	2.41 ± 0.74 ^c^	68.67 ± 2.21 ^d^	0.79 ± 0.03 ^d^
S_0.5_	117.51 ± 16.86 ^c^	24.34 ± 0.75 ^b^	1.12 ± 0.06 ^b^	64.76 ± 4.11 ^d^	0.61 ± 0.14 ^c^
S_1_	72.17 ± 9.55 ^b^	20.41 ± 1.61 ^a^	1.11 ± 0.07 ^b^	45.21 ± 2.35 ^c^	0.55 ± 0.18 ^bc^
S_2_	71.46 ± 9.74 ^b^	20.67 ± 1.14 ^a^	0.50 ± 0.10 ^ab^	41.35 ± 2.36 ^c^	0.37 ± 0.02 ^ab^
S_5_	37.12 ± 7.11 ^a^	19.29 ± 0.68 ^a^	0.11 ± 0.01 ^a^	15.87 ± 1.09 ^a^	0.32 ± 0.03 ^a^
S_10_	47.26 ± 9.92 ^a^	21.82 ± 0.64 ^ab^	0.06 ± 0.02 ^a^	30.91 ± 6.07 ^b^	0.39 ± 0.12 ^ab^

S_0_, S_0.25_, S_0.5_, S_1_, S_2_, S_5_, and S_10_ represent succinylated EY obtained at the succinic anhydride addition of 0, 0.25%, 0.5%, 1%, 2%, 5%, and 10% (*w*/*w*), respectively. ^a, b, c, d^ Different letters in the same series indicate statistically significant differences (means ± standard deviation, *n* = 3, *p* < 0.05).

**Table 2:** Oil exudation, TBARS, and carbonyl concentration of succinylated egg yolk.

	Oil Exudation (%)		TBARS (mmol MDA/ mg Homogenate)		Carbonyls (nmol/mg Protein)	
	65 °C	70 °C	65 °C	70 °C	65 °C	70 °C
S_0_	1.46 ± 0.10 ^c^	2.72 ± 0.14 ^c^	1.88 ± 0.12 ^c^	2.44 ± 0.11 ^c^	0.52 ± 0.05 ^c^	0.73 ± 0.05 ^c^
S_0.5_	0.50 ± 0.09 ^b^	0.68 ± 0.06 ^b^	1.52 ± 0.10 ^b^	1.77 ± 0.24 ^b^	0.39 ± 0.06 ^b^	0.63 ± 0.03 ^b^
S_5_	0.33 ± 0.04 ^a^	0.48 ± 0.05 ^a^	1.22 ± 0.17 ^a^	1.27 ± 0.08 ^a^	0.31 ± 0.02 ^a^	0.40 ± 0.03 ^a^

Succinylated egg yolk heated at 65 °C and 70 °C, respectively. S_0_, S_0.5_, and S_5_ represent succinylated EY obtained at the anhydride addition of 0, 0.5, and 5 (*w*/*w*), respectively. ^a, b, c^ Different letters in the same series indicate statistically significant differences (means ± standard deviation, *n* = 3, *p* < 0.05).

**Table 3:** The percentage of β-sheet, α-helix, β-turn, and random coil of proteins of LDL particles in succinylated egg yolk.

	Content of Secondary Structures (%)			
	β-Sheets	α-Helices	β-Turns	Random Coils
S_0_	22.60 ± 1.01 ^c^	30.92 ± 0.58 ^a^	29.65 ± 0.74 ^ab^	16.83 ± 0.30 ^b^
S_0.25_	21.91 ± 3.08 ^c^	29.54 ± 1.23 ^a^	33.81 ± 1.97 ^a^	14.75 ± 0.11 ^ab^
S_0.5_	20.87 ± 1.89 ^bc^	28.35 ± 4.38 ^a^	31.40 ± 3.48 ^ab^	19.38 ± 0.99 ^c^
S_1_	18.25 ± 2.17 ^abc^	31.20 ± 6.06 ^a^	33.48 ± 1.87 ^b^	17.06 ± 2.02 ^b^
S_2_	18.82 ± 1.65 ^abc^	32.96 ± 2.30 ^ab^	31.37 ± 0.05 ^ab^	16.84 ± 0.70 ^b^
S_5_	16.41 ± 2.73 ^ab^	39.69 ± 2.71 ^bc^	29.01 ± 0.51 ^a^	14.89 ± 0.49 ^ab^
S_10_	13.77 ± 0.89 ^a^	42.64 ± 0.05 ^c^	29.44 ± 0.68 ^ab^	14.15 ± 0.15 ^a^

S_0_, S_0.25_, S_0.5_, S_1_, S_2_, S_5_, and S_10_ represent succinylated EY obtained at the anhydride addition of 0, 0.25%, 0.5%, 1%, 2%, 5%, and 10% (*w*/*w*), respectively. ^a, b, c^ Different letters in the same series indicate statistically significant differences (means ± standard deviation, *n* = 2, *p* < 0.05).

## Data Availability

The original contributions presented in the study are included in the article; further inquiries can be directed to the corresponding author.
